# Early Prediction of the Evolution of Self‐Limited Epilepsy With Centrotemporal Spikes to Epileptic Encephalopathy With Spike‐and‐Wave Activation in Sleep: A Prediction Model Construction Based on Quantitative Electroencephalography Characteristics

**DOI:** 10.1111/cns.70268

**Published:** 2025-03-18

**Authors:** Zimeng He, Linghui Zhu, Zaifen Gao, Yumei Li, Xiaoyu Zhao, Xiaofan Yang, Lili Tong, Guijuan Jia, Dongqing Zhang, Baomin Li

**Affiliations:** ^1^ Shandong University Jinan Shandong China; ^2^ Department of Pediatrics Qilu Hospital of Shandong University Jinan Shandong China; ^3^ Department of Epilepsy Center Children's Hospital Affiliated to Shandong University, Jinan Children's Hospital Jinan Shandong China

**Keywords:** epileptic encephalopathy with spike‐and‐wave activation in sleep (EE‐SWAS), functional connectivity, prediction model, quantitative electroencephalography (qEEG), self‐limited epilepsy with centrotemporal spikes (SeLECTS)

## Abstract

**Aims:**

To predict the progression of children with self‐limited epilepsy with centrotemporal spikes (SeLECTS) to epileptic encephalopathy with spike‐and‐wave activation in sleep (EE‐SWAS).

**Methods:**

We conducted a retrospective analysis of early clinical and electroencephalography (EEG) data. Clinical parameters included demographic and epilepsy‐related characteristics. EEG were qualitatively (localization, lateralization, synchrony, non‐Rolandic discharges, nondipole spikes, multiple spikes, focal slow‐wave activity) and quantitatively (spike–wave index [SWI], spike–wave frequency [SWF], power spectral density [PSD], phase‐locking value [PLV], phase lag index [PLI], weighted phase lag index [wPLI], characteristic path length [CPL], clustering coefficient [CC], small‐worldness [Sigma]) analyzed. A logistic regression‐based prediction model was further formulated and evaluated.

**Results:**

This study included 50 children with seizure‐free typical SeLECTS and 76 who developed EE‐SWAS. Multivariable logistic regression revealed that early EEG features—SWF, relative PSD in the alpha band, wPLI and CPL in the delta band—were associated with the risk of encephalopathic transformation. The model demonstrated good performance with an area under the curve of 0.817 (95% confidence interval 0.736–0.898). The model showed a good fit and clinical benefit.

**Conclusion:**

Initial quantitative EEG characteristics of SeLECTS can predict the development of EE‐SWAS, suggesting distinct disease characteristics and pathogeneses in children at risk of encephalopathic transformation.

## Graphical abstract

In children with SeLECTS, early EEG features such as SWF, relative PSD, wPLI, and CPL are significantly associated with the presence of EE‐SWAS during the disease course. We successfully construct a model to predict the evolution of EE‐SWAS in SeLECTS, thereby aiding early clinical diagnosis and timely intervention for EE‐SWAS.

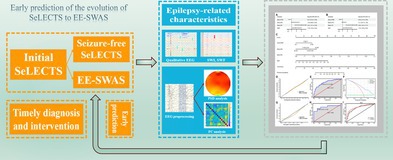

## 1 Introduction

Self‐limited epilepsy with centrotemporal spikes (SeLECTS) is the most common self‐limited focal epilepsy in children, accounting for 6%–7% of all childhood epilepsies [1]. Formerly known as benign epilepsy with centrotemporal spikes, SeLECTS generally follows a benign course. Most affected children experience only a few seizures, respond well to anti‐seizure medications (ASMs), and typically see seizure resolution by the age of 12 or 13 [2]. Additionally, some children with SeLECTS may exhibit comorbid neuropsychological impairments in language, memory, and attention [3], some of which may improve or resolve by adulthood; however, a considerable number of patients still have a variety of adverse social outcomes, including depression or another psychiatric diagnosis, educational issues, social difficulties, and poverty [4, 5].

Despite its generally favorable prognosis, SeLECTS can atypically progress to epileptic encephalopathy with spike‐and‐wave activation during sleep (EE‐SWAS), affecting 1.3%–4.6% of cases [6]. EE‐SWAS is characterized by frequent spike‐and‐wave activation during sleep periods on the EEG which was known as electrical status epilepticus in sleep (ESES), and is associated with a spectrum of developmental regressions including cognitive, linguistic, behavioral, and motor skills [1]. While clinical seizures and the ESES pattern on EEG typically resolve by puberty, the associated neurocognitive or motor impairments may persist permanently in about half of the patients [7, 8]. The duration and etiology of EE‐SWAS are key determinants of long‐term prognosis [9]. Although previous studies have recognized and described the evolution from SeLECTS to EE‐SWAS [10, 11, 12], diagnostic delays are common due to the absence of early predictive markers. EE‐SWAS is often only identified following an exacerbation of seizures or the emergence of the ESES pattern on EEG, resulting in delayed treatment and irreversible neurocognitive deterioration.

Over the past decade, quantitative EEG (qEEG) analysis techniques have become increasingly common in the field of epilepsy research. Recently, several studies have looked at the power and functional connectivity (FC) of SeLECTS using resting‐state EEG or magnetoencephalography (MEG) data. In a case–control study based on SeLECTS patients with clinical seizure remission and healthy participants, qEEG analysis revealed a significant increase in the absolute power of the theta and alpha waves in SeLECTS patients [13]. Two EEG studies revealed a significant increase in connectivity during spike discharges: A study by Ghantasala et al. showed significantly higher coherence in the theta, alpha and beta bands in EEG epochs with centrotemporal spikes compared to epochs without spikes [14]; Goad et al. [15] found that children with SeLECTS had significantly higher average weighted phase lag index values in most electrodes than controls during sleep. A study based on EEG graph‐theoretical analysis further showed that global efficiency, mean clustering coefficient, and mean nodal strength at the whole‐brain level were significantly increased in patients with SeLECTS compared to controls [16]. This series of studies suggested that children with SeLECTS may have underlying brain dysfunction or delayed maturation related to neurocognition, which can be reflected by differences in power and FC [13, 17]. We hypothesized that using qEEG analysis to explore potential differences in power and FC at the onset of SeLECTS might help determine whether SeLECTS will develop into EE‐SWAS. It is worth noting that previous studies had mostly focused on SeLECTS and healthy populations, and this approach has not been studied in the SeLECTS population undergoing encephalopathic transformation. Consequently, our research aims to develop a prediction model focusing on EEG indicators at the onset of SeLECTS including qualitative and quantitative characteristics, enabling the early detection of EE‐SWAS and prompt initiation of interventions to maximize the benefits of early treatment [11].

## 2 Methods

### 2.1 Study Design and Participants

This retrospective study included children diagnosed with SeLECTS or EE‐SWAS between January 2016 and January 2022 at three hospitals: Qilu Hospital of Shandong University, Jinan Children's Hospital, and Jinan Minzu Hospital. The local ethics committee approved the study (Ethics approval number: KYLL‐202403‐043). All participants were initially diagnosed with SeLECTS. Based on the clinical course of disease, we categorized the children into two groups: Typical SeLECTS and EE‐SWAS group. We used a spike–wave index (SWI) of 50% during non‐rapid eye movement (NREM) sleep as the threshold to differentiate EEG patterns between SeLECTS and EE‐SWAS [18]. The specific inclusion criteria were shown below: Typical SeLECTS: (1) Patients met the 2022 International League Against Epilepsy (ILAE) diagnostic criteria for SeLECTS [1] (EEG showing SWI < 50% during NREM sleep, with normal cognition and development); (2) Seizure‐free for more than 2.5 years during follow‐up; (3) The entire epilepsy course lasted 3–6 years, during which time EEGs were performed every 3–6 months, none of which showed ESES; (4) There was no developmental regression during the course of the disease; (5) Availability of raw EEG data from early disease onset. EE‐SWAS: (1) Patients who initially diagnosed with SeLECTS developed EE‐SWAS fulfilling the 2022 ILAE criteria (EEG showing SWI ≥ 50% during NREM sleep with temporally related cognitive or behavioral regression); (2) Patients underwent the neurodevelopmental assessment of China‐Wechsler Intelligence Scale for Children (C‐WISC), and EE‐SWAS was diagnosed only if language or cognitive impairment was present; (3) Availability of raw EEG data from early disease onset. Among all children initially diagnosed with SeLECTS, we excluded those whose initial EEGs displayed a SWI ≥ 50%, regardless of normal cognitive and behavioral assessments, to prevent potential bias in participant selection for this study. Data from Qilu Hospital of Shandong university and Jinan Children's Hospital formed the training cohort for developing the prediction model, while data from Jinan Minzu Hospital were utilized for external validation.

### 2.2 Data Collection and Analysis

#### 2.2.1 Clinical Data

Demographic and clinical data from the early stages (within 1 year of the first‐ever seizure) of SeLECTS were collected through medical record reviews and telephone follow‐ups. Analyzed clinical indicators included sex, age at SeLECTS onset, age at EEG recording, seizure semiology (focal seizures, focal to bilateral tonic–clonic seizures, or both), seizure frequency (within 3 and 12 months), occurrence of daytime seizures, seizure duration more than 5 min, anti‐seizure medication (ASM) use (monotherapy or polytherapy), history of febrile seizures (FS), family history of epilepsy, comorbid attention‐deficit/hyperactivity disorder (ADHD), genetic test results, and age at EE‐SWAS diagnosis.

#### 2.2.2 EEG Data

EEG results from each participant following the initial seizure were retrospectively collected. Qualitative EEG analysis was performed by two neurophysiologists; any disagreements were discussed until a consensus was reached. Additionally, raw EEG data were gathered for further quantitative analysis. EEG recordings were sampled at 500 or 1000 Hz, including sleep periods of at least 30 min. The 10–20 electrode placement system was used for EEGs, and only the following electrodes were included for quantitative EEG analysis: Fp1, Fp2, F3, F4, F7, F8, C3, C4, T3, T4, T5, T6, P3, P4, O1, O2, Fz, Cz, Pz. All raw EEG data were quantitatively analyzed using Matlab R2013b.

##### 2.2.2.1 Qualitative EEG Analysis

The assessment included the presence or absence of the following EEG features: interictal epileptiform discharges (IEDs) outside the Rolandic region, lateralization of IEDs (unilateral or bilateral), synchrony of IEDs (synchronous or asynchronous), localization of spike‐and‐wave abnormalities (anterior or posterior), nondipole spikes, Rolandic multiple spikes, and focal slow‐wave activity. When EEGs showed bilateral spike focus, discharges were further classified as symmetrical or asymmetrical. A temporal‐frontal dipole spike is defined as a discharge exhibiting temporoparietal negativity and simultaneous frontal positivity. If a dipole field is present in less than 80% of discharges, the spikes are classified as nondipole [19]. Focal slow wave activity is defined as focal slowing observable in the same region as spikes, distinct from slow waves accompanying the spikes in spike‐and‐slow‐wave complexes [20].

##### 2.2.2.2 Spike–Wave Index (SWI) and Spike–Wave Frequency (SWF)

In this study, the SWI was calculated by determining the percentage of spike‐and‐wave activity during the first 5 min of NREM sleep [21]. This was achieved by dividing the number of seconds containing spike‐and‐wave activity by 300 s and then multiplying by 100. The SWF was determined by calculating the average frequency of spike–wave activities per 100 s within the 5‐min period of the EEG [22]. Both SWI and SWF were calculated through visual inspection by two neurophysiologists.

##### 2.2.2.3 EEG Preprocessing and Power Spectral Density (PSD) Analysis

Raw EEG data were processed using a band‐pass filter set to 1–40 Hz and resampled at 500 Hz. Neurophysiologists conducted manual visual inspections to identify and remove all artifacts/electrical noises. Independent component analysis (ICA), using the Runica algorithm from the EEGlab toolbox, was employed to exclude contaminated EEG epochs [23]. For each patient, a continuous 5‐min segment of EEG was selected from artifact‐free interictal EEGs (containing at least a 15‐min stage 2 NREM period in the first sleep cycle). The selected 5‐min EEG was further epoched into a series of 2‐s epochs and re‐referenced based on the common average reference method. PSD was calculated for these 5‐min EEG epochs based on Welch's method using the “pwelch” function from EEGlab (2‐s window, 50% overlap). For each patient, both absolute PSD (μV^2^) and relative PSD (%) were calculated across four frequency bands: delta (1–4 Hz), theta (4–8 Hz), alpha (8–13 Hz), and beta (13–30 Hz) frequency bands. The mean values of power were averaged across all electrodes (whole‐brain average). Relative PSD was determined by dividing the absolute power of a specific frequency band by the total power, enhancing comparability between subjects [24].

#### 2.2.3 Functional Connectivity (FC) Analysis

To avoid the impact of volume conduction on FC analysis, we applied a current source density (CSD) transformation to the raw EEG data using the CSD toolbox [25] to eliminate common source issues. For each preprocessed 5‐min segment of EEG data (preprocessing procedure is as described above), a Hilbert transform was performed to obtain the instantaneous phase time‐series, which were used to calculate the FC metrics for each electrode pair. The values of FC metrics are calculated for each 2‐s epoch, and then averaged across all epochs. The mean connectivity across all electrode pairs in each frequency band was calculated to obtain a single coupling value, referred to as the global value.

##### 2.2.3.1 Synchronization‐Based FC Metrics

Phase‐locking value (PLV), phase lag index (PLI), and weighted phase lag index (wPLI) were calculated separately to assess functional connectivity. The PLV, which ranges from 0 to 1, measures the phase difference between different signals [26], with 0 indicating unsynchronized phases and 1 indicating perfect synchronization. The PLI evaluates the asymmetry in the distribution of phase differences between signals [27], with values also ranging from 0 to 1, where higher values indicate greater connectivity. The wPLI, which is based on the imaginary component of the cross‐spectrum, quantifies non‐zero phase difference synchronization between signals [28] and improves the robustness to noise [29]. We calculated the source‐level PLV values using the Fieldtrip toolbox (multi‐taper method fast Fourier transform, single Hanning taper, 1 Hz frequency resolution) to exclude the impact of volume conduction on the PLV. The global values of PLV, PLI, and wPLI were obtained by averaging the values of these metrics across all electrode pairs.

##### 2.2.3.2 Functional Network Construction and Graph‐Theoretical Metrics Calculation

A weighted undirected graph was constructed with 19 EEG electrodes as nodes, and the source‐level PLV values between electrode pairs defined as edges. We calculated the following metrics for each patient in each frequency band using the GRETNA toolbox [30]: characteristic path length (CPL), clustering coefficient (CC), and small‐worldness (Sigma). The CPL is the average of the shortest path lengths between all node pairs and characterizes the brain's ability to integrate information from distributed areas [31]. The CC quantifies the intensity of the neighbors of connected nodes in the network, reflecting the brain's capacity to process information within interconnected clusters [31]. A network exhibits small‐world properties if it optimally balances the segregation and integration of information [32]. The small‐worldness (Sigma) quantifies these properties by calculating from CC and CPL, with a Sigma > 1 indicating small‐world characteristics [33]. For graph‐theoretical metrics, different choices of sparsity correspond to different values. We calculated each network metrics by computing the area under the curve (AUC) across different sparsities (sparsity range 0.1–0.5, step length 0.05) and normalized them to obtain their true values [34].

#### 2.2.4 Statistical Analysis and Model Development

Data analyses were conducted using IBM SPSS Statistics 26.0 (Armonk, New York, USA). The normality of data was assessed with the Shapiro–Wilk test. Normally distributed continuous variables were presented as mean ± standard deviation (SD) and analyzed using the independent samples *t*‐test. Non‐normally distributed continuous variables were expressed as median ± interquartile range (IQR) and analyzed with the non‐parametric Mann–Whitney *U* test. Categorical variables, expressed as numbers and percentages, were compared using chi‐square (*χ* ^2^) or Fisher's exact tests. Some continuous variables were converted into categorical variables based on IQR for further analysis. For group comparisons across all qualitative and quantitative EEG characteristics, the Benjamini‐Hochberg false discovery rate (FDR) procedure was applied to correct the *p* values [35]. Univariable logistic regression was performed for included variables, and those with FDR‐corrected *p* value < 0.20 were selected for multivariable analysis. Multivariable logistic regression with backward stepwise variable selection was performed, including factors with statistical significance in the prediction model. The variance inflation factor (VIF) coefficients were examined to avoid collinearity among the variables. All tests were two‐tailed, and a *p* value < 0.05 was considered statistically significant.

The final prediction model was constructed using R 4.3.3 (R Foundation for Statistical Computing, Vienna, Austria). A nomogram was created based on the predictors in the model. The receiver operating characteristic (ROC) curve was used to evaluate model discrimination, with the prediction performance quantified by the AUC. The calibration curve and Hosmer–Lemeshow test assessed goodness‐of‐fit, with a *p* value > 0.05 indicating good calibration. Decision curve analysis (DCA) estimated the clinical benefits by calculating the net benefits of threshold probabilities. Internal validation was performed using the bootstrapping method (1000 bootstrap samples) to assess model stability.

## 3 Results

### 3.1 Demographics and Clinical Characteristics

A total of 126 children initially diagnosed with SeLECTS were included in the study (Of all SeLECTS children who developed EE‐SWAS, 11 were excluded for having an initial SWI > 50%; of all SeLECTS children without encephalopathic transformation, 2 were excluded for having an initial SWI > 50%). Of all the children included, 106 were used to build the prediction model (training cohort), and 20 were used for external validation (validation cohort). In the training cohort, 64 children developed EE‐SWAS, and 42 did not, classified as the EE‐SWAS and SeLECTS groups, respectively. The mean age at SeLECTS onset in the 106 children was 7.61 ± 1.59 years. The mean age at EEG recording was 7.73 ± 1.62 years, and the median interval between the SeLECTS diagnosis and EEG recording was 1 [0–2] month. The mean age at EE‐SWAS diagnosis was 9.08 ± 1.29 years, with a mean interval of 1.58 ± 0.72 years between SeLECTS diagnosis and EE‐SWAS onset. One child in the SeLECTS group and two children in the EE‐SWAS group had used ASM before the EEG recording. In the training cohort, genetic testing was performed on 21 patients in the EE‐SWAS group, and pathogenic or likely pathogenic gene mutations were detected in four of them, including genes *SCN1A*, *SCN2A*, *GRIN2A*, and *ARHGEF9*; genetic testing was performed on 9 patients in the SeLECTS group, and a pathogenic mutation of *GRIN2A* gene was detected in only one of them, while no other pathogenic variants were found. Demographic and clinical characteristics of both groups are detailed in Table 1, showing no significant differences between the two groups.

**TABLE 1:** Demographic and clinical characteristics for SeLECTS and EE‐SWAS groups.

Demographic and clinical characteristics	EE‐SWAS (*n* = 64)	SeLECTS (*n* = 42)	*p*
Male, *n* (%)	30 (46.9)	24 (57.1)	0.301^a^
Age at SeLECTS onset, years (mean ± SD)	7.50 ± 1.47	7.79 ± 1.77	0.363^b^
Seizure semiology			
Focal seizures, *n* (%)	16 (25.0)	10 (23.8)	0.893^c^
Focal to BTCS, *n* (%)	43 (67.2)	30 (71.4)	
Mixed, *n* (%)	5 (7.8)	2 (4.8)	
Number of seizures			
Within the first year^d^, *n* (median [P25–P75])	3 [2–5]	3 [2–5]	0.805^e^
Within the first 3 months, *n* (median [P25–P75])	2 [1–2]	1.5 [1–3]	0.795^e^
Seizure frequency in the first year^d^			
> 4 seizures, *n* (%)	15 (30.0)	12 (28.6)	0.881^a^
≤ 4 seizures, *n* (%)	35 (70.0)	30 (71.4)	
Daytime seizures, *n* (%)	6 (9.4)	7 (16.7)	0.263^a^
Seizure duration more than 5 min, *n* (%)	17 (26.6)	9 (21.4)	0.548^a^
Family history of epilepsy, *n* (%)	6 (9.4)	4 (9.5)	1.000^a^
History of febrile seizures, *n* (%)	6 (9.4)	6 (14.3)	0.640^a^
Comorbid ADHD within the first year of onset^f^			
With comorbid ADHD, *n* (%)	8 (21.6)	2 (11.1)	0.565^a^
Without comorbid ADHD, *n* (%)	29 (78.4)	16 (88.9)	
Number of ASMs before EE‐SWAS			
Non‐use of ASMs, *n* (%)	2 (3.2)	3 (7.1)	0.389^c^
Monotherapy, *n* (%)	39 (60.9)	28 (66.7)	
Polytherapy, *n* (%)	23 (35.9)	11 (26.2)	
Use of ASMs before EE‐SWAS			
Valproic acid, *n* (%)	23 (35.9)	9 (21.4)	0.111^a^
Levetiracetam, *n* (%)	34 (53.1)	22 (52.4)	0.940^a^
Oxcarbazepine, *n* (%)	12 (18.8)	10 (23.8)	0.530^a^
Benzodiazepines (CZP, CLB), *n* (%)	5 (7.8)	2 (4.8)	0.827^a^
Other (LCM, LTG, CBZ, PER, TPM, ZNS, PB), *n* (%)	12 (18.8)	7 (16.7)	0.784^a^
Use of sodium channel blockers^g^, *n* (%)	16 (25.0)	11 (26.2)	0.891^a^
Age at EEG recording, years (mean ± SD)	7.63 ± 1.49	7.89 ± 1.81	0.419^b^
Age at EE‐SWAS diagnosis, years (mean ± SD)	9.08 ± 1.29		
Time between diagnosis of SeLECTS and EE‐SWAS, years (mean ± SD)	1.58 ± 0.72		

Abbreviations: ADHD, attention‐deficit/hyperactivity disorder; ASMs, anti‐seizure medications; BTCS, bilateral tonic–clonic seizures; CBZ, carbamazepine; CLB, clobazam; CZP, clonazepam; EEG, electroencephalogram; EE‐SWAS, epileptic encephalopathy with spike‐and‐wave activation in sleep; LCM, lacosamide; LTG, lamotrigine; PB, phenobarbitone; PER, perampanel; SD, standard deviation; SeLECTS, self‐limited epilepsy with centrotemporal spikes; TPM, topiramate; ZNS, zonisamide.

^a^ *p* value was calculated using Chi‐squared test.

^b^ *p* value was calculated using independent *t*‐test.

^c^ *p* value was calculated using Fisher exact test.

^d^ When comparing the number of seizures or seizure frequency within the first year, children in the EE‐SWAS group with a time from diagnosis of SeLECTS to EE‐SWAS of less than 1 year were excluded.

^e^ *p* value was calculated using Mann–Whitney *U* test.

^f^ Only children who underwent the ADHD‐related assessment and were diagnosed with ADHD based on Diagnostic and Statistical Manual of Mental Disorders‐IV/V (DSM‐IV/V) diagnostic criteria were included.

^g^ Sodium channel blockers include oxcarbazepine, carbamazepine, lamotrigine, and lacosamide.

### 3.2 Qualitative‐EEG Characteristics, SWI, and SWF

**FIGURE 1:**
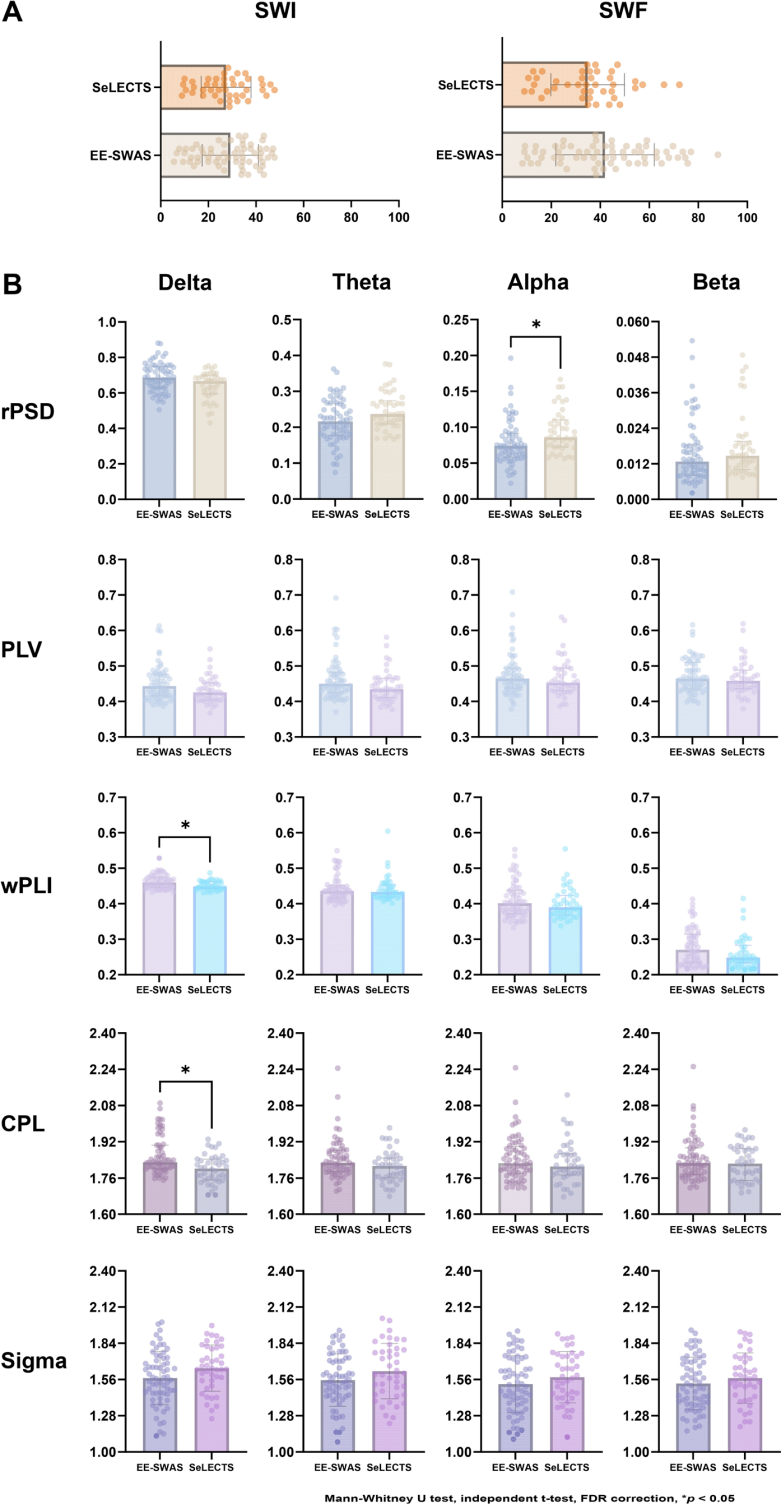
Bar graphs of variables with significant differences between SeLECTS and EE‐SWAS groups. (A) Differences in SWI and SWF value between SeLECTS and EE‐SWAS groups. The SWF value was higher in EE‐SWAS group than that in SeLECTS group, although the difference was not significant (uncorrected *p* = 0.040, FDR‐corrected *p* = 0.164). (B) Differences in mean values of rPSD, PLV, wPLI, CPL, and Sigma between SeLECTS and EE‐SWAS groups in different frequency bands. The rPSD value in the EE‐SWAS group was significantly lower in the alpha band (FDR‐corrected *p* = 0.048). The global wPLI value in the delta band was significantly higher for the EE‐SWAS group (FDR‐corrected *p* = 0.048). The CPL value in the delta band was significantly longer for the EE‐SWAS group (FDR‐corrected *p* = 0.022). The values of PLV and Sigma showed no significant differences between the groups across all frequency bands. Asterisks indicate significant differences between SeLECTS and EE‐SWAS groups (Mann–Whitney *U* test, independent *t*‐test, FDR correction, *p < 0.05). Abbreviations: CPL, characteristic path length; EE‐SWAS, epileptic encephalopathy with spike‐and‐wave activation in sleep; FDR, false discovery ratePLV, phase‐locking value; PSD, power spectral density; SeLECTS, self‐limited epilepsy with centrotemporal spikes; Sigma, small‐worldness; SWF, spike–wave frequency; SWI, spike–wave index; wPLI, weighted phase lag index.

The qualitative EEG characteristics and the values of SWI and SWF for the two groups are presented in Table 2. No significant differences were found between the two groups in terms of localization, lateralization, and synchrony of EEG, non‐Rolandic discharges, nondipole spikes, Rolandic multiple spikes, focal slow‐wave activity, and SWI value. Notably, the SWF value for the EE‐SWAS group was higher than that of the SeLECTS group (uncorrected *p* = 0.040). Although the differences between groups after FDR correction are not statistically significant, they are still valuable considering the relative stringency of the FDR method. In fact, the SWF has been proved to have predictive value according to subsequent regression analysis. Figure 1A illustrates the differences in SWI and SWF between the groups.

**TABLE 2:** Qualitative‐EEG characteristics, SWI, and SWF for SeLECTS and EE‐SWAS groups.

EEG characteristics	EE‐SWAS (*n* = 64)	SeLECTS (*n* = 42)	*p* (Uncorrected)	*p* (FDR‐corrected)
IEDs outside the Rolandic region, *n* (%)	31 (48.4)	16 (38.1)	0.294	0.480
Lateralization of IEDs			0.568	0.674
Unilateral, *n* (%)	18 (28.1)	14 (33.3)	0.265	0.480
Left, *n* (%)	10 (15.6)	5 (11.9)		
Right, *n* (%)	8 (12.5)	9 (21.4)		
Bilateral, *n* (%)	46 (71.9)	28 (66.7)	0.582	0.674
Symmetrical, *n* (%)	9 (14.1)	7 (16.7)		
Asymmetrical, *n* (%)	37 (57.8)	21 (50.0)		
Synchrony of IEDs				
Synchronous, *n* (%)	38 (59.4)	25 (59.5)	0.988	0.988
Asynchronous, *n* (%)	26 (40.6)	17 (40.5)		
Localization of Rolandic spikes				
Anterior, *n* (%)	36 (56.2)	22 (52.4)	0.695	0.746
Posterior, *n* (%)	28 (43.8)	20 (47.6)		
Nondipole spikes, *n* (%)	44 (68.8)	27 (64.3)	0.633	0.696
Rolandic multiple spikes, *n* (%)	19 (29.7)	7 (16.7)	0.128	0.313
Focal slow‐wave activity, *n* (%)	9 (14.1)	6 (14.3)	0.974	0.988
SWI value, % (mean ± SD)	29.3 ± 11.8	27.4 ± 10.5	0.418	0.541
SWF value, % (mean ± SD)	41.9 ± 20.1	34.9 ± 15.0	0.040	0.164

*Note:* Asterisks indicate significant differences between SeLECTS and EE‐SWAS groups (Chi‐squared test, independent *t*‐test, FDR correction, *p* < 0.05).

Abbreviations: EEG, electroencephalogram; EE‐SWAS, epileptic encephalopathy with spike‐and‐wave activation in sleep; FDR, false discovery rate; IEDs, interictal epileptiform discharges; SD, standard deviation; SeLECTS, self‐limited epilepsy with centrotemporal spikes; SWF, spike–wave frequency; SWI, spike–wave index.

### 3.3 Absolute and Relative PSD

Age and sex influence the power and frequency of EEG. In our study, the sex composition and age at EEG recording were comparable between the two groups. The values of absolute and relative PSD in each frequency band are shown in Table 3. For the absolute PSD, there were no significant differences between groups in all frequency bands. The relative PSD in the EE‐SWAS group was significantly lower in the alpha bands (FDR‐corrected *p* = 0.048), and no significant differences between groups were observed in other bands. Figure 1B illustrates the differences in power between the groups.

**TABLE 3:** Absolute PSD and relative PSD in different frequency bands for SeLECTS and EE‐SWAS groups.

Band (whole‐brain)^a^	Absolute PSD (μV^2^)				Relative PSD (%)			
	EE‐SWAS (*n* = 64)	SeLECTS (*n* = 42)	*p* (Uncorrected)	*p* (FDR‐corrected)	EE‐SWAS (*n* = 64)	SeLECTS (*n* = 42)	*p* (Uncorrected)	*p* (FDR‐corrected)
Full‐frequency band	63.48 ± 84.69	35.18 ± 29.46	0.017	0.125				
Delta	42.41 ± 63.26	25.10 ± 21.43	0.010	0.110	68.73 ± 12.05	66.71 ± 10.10	0.013	0.114
Theta	12.54 ± 17.69	8.18 ± 6.26	0.109	0.285	21.63 ± 8.96	23.67 ± 6.49	0.027	0.164
Alpha	3.90 ± 5.56	3.41 ± 2.72	0.314	0.480	7.41 ± 3.35	8.61 ± 3.77	0.004	0.048*
Beta	0.68 ± 0.72	0.57 ± 0.36	0.208	0.420	1.28 ± 1.04	1.47 ± 0.95	0.152	0.345

*Note:* Data are presented as median ± interquartile range (IQR).

Abbreviations: EE‐SWAS, epileptic encephalopathy with spike‐and‐wave activation in sleep; FDR, false discovery rate; PSD, power spectral density; SeLECTS, self‐limited epilepsy with centrotemporal spikes.

^a^ Whole‐brain average is an average of the power values of all electrodes.

* Significant differences between SeLECTS and EE‐SWAS groups (Mann–Whitney *U* test, FDR correction, *p* < 0.05).

### 3.4 FC Analysis and Graph‐Theoretical Metrics

The values of synchronization‐based FC and graph‐theoretical metrics for each frequency band are shown in Table 4. FC analysis showed that the global wPLI in the delta band was significantly higher for the EE‐SWAS group (FDR‐corrected *p* = 0.048). The PLV and PLI showed no significant differences between the groups. Graph‐theoretical analysis revealed that the CPL in the delta band was significantly longer in the EE‐SWAS group compared to the SeLECTS group (FDR‐corrected *p* = 0.022). The CC and Sigma showed no significant differences between the groups. Figure 1B further illustrates the differences in FC metrics between the groups.

**TABLE 4:** The values of synchronization‐based functional connectivity and graph‐theoretical metrics in different frequency bands for SeLECTS and EE‐SWAS group.

Metric value	Delta			Theta			Alpha			Beta		
	EE‐SWAS	SeLECTS	*p*	EE‐SWAS	SeLECTS	*p*	EE‐SWAS	SeLECTS	*p*	EE‐SWAS	SeLECTS	*p*
Global PLV	0.443 ± 0.061	0.425 ± 0.049	0.164	0.450 ± 0.057	0.435 ± 0.055	0.164	0.465 ± 0.056	0.453 ± 0.063	0.495	0.464 ± 0.075	0.458 ± 0.052	0.575
Global PLI	0.287 ± 0.018	0.284 ± 0.014	0.444	0.265 ± 0.018	0.262 ± 0.015	0.694	0.224 ± 0.022	0.220 ± 0.023	0.285	0.130 ± 0.010	0.130 ± 0.009	0.345
Global wPLI	0.460 ± 0.029	0.449 ± 0.019	0.048*	0.436 ± 0.036	0.433 ± 0.033	0.923	0.402 ± 0.066	0.390 ± 0.056	0.495	0.271 ± 0.080	0.249 ± 0.049	0.217
CPL^a^	1.830 ± 0.104	1.802 ± 0.092	0.022*	1.828 ± 0.095	1.813 ± 0.088	0.164	1.826 ± 0.120	1.811 ± 0.094	0.480	1.826 ± 0.117	1.824 ± 0.140	0.541
CC^a^	0.663 ± 0.022	0.661 ± 0.022	0.672	0.665 ± 0.019	0.659 ± 0.022	0.285	0.664 ± 0.018	0.660 ± 0.021	0.480	0.667 ± 0.022	0.663 ± 0.021	0.495
Sigma^a^	1.572 ± 0.206	1.649 ± 0.179	0.164	1.556 ± 0.203	1.625 ± 0.214	0.285	1.524 ± 0.220	1.578 ± 0.198	0.420	1.529 ± 0.203	1.571 ± 0.195	0.480

*Note:* CC and Sigma are presented as mean ± standard deviation (SD), other data are presented as median ± interquartile range (IQR). Only false discovery rate (FDR)‐corrected *p* values are shown in Table 4.

Abbreviations: CC, clustering coefficient; CPL, characteristic path length; EE‐SWAS, epileptic encephalopathy with spike‐and‐wave activation in sleep; PLI, phase lag index; PLV, phase‐locking value; SeLECTS, self‐limited epilepsy with centrotemporal spikes; Sigma, small‐worldness; wPLI, weighted phase lag index.

^a^ The graph‐theoretical metrics were calculated by computing the area under the curve (AUC) across different sparsities (sparsity range 0.1–0.5, step length 0.05) and further normalized to obtain their true values.

* Significant differences between SeLECTS and EE‐SWAS groups (Mann–Whitney *U* test, independent *t*‐test, FDR correction, *p* < 0.05).

### 3.5 Construction and Performance Evaluation of the Prediction Model

**FIGURE 2:**
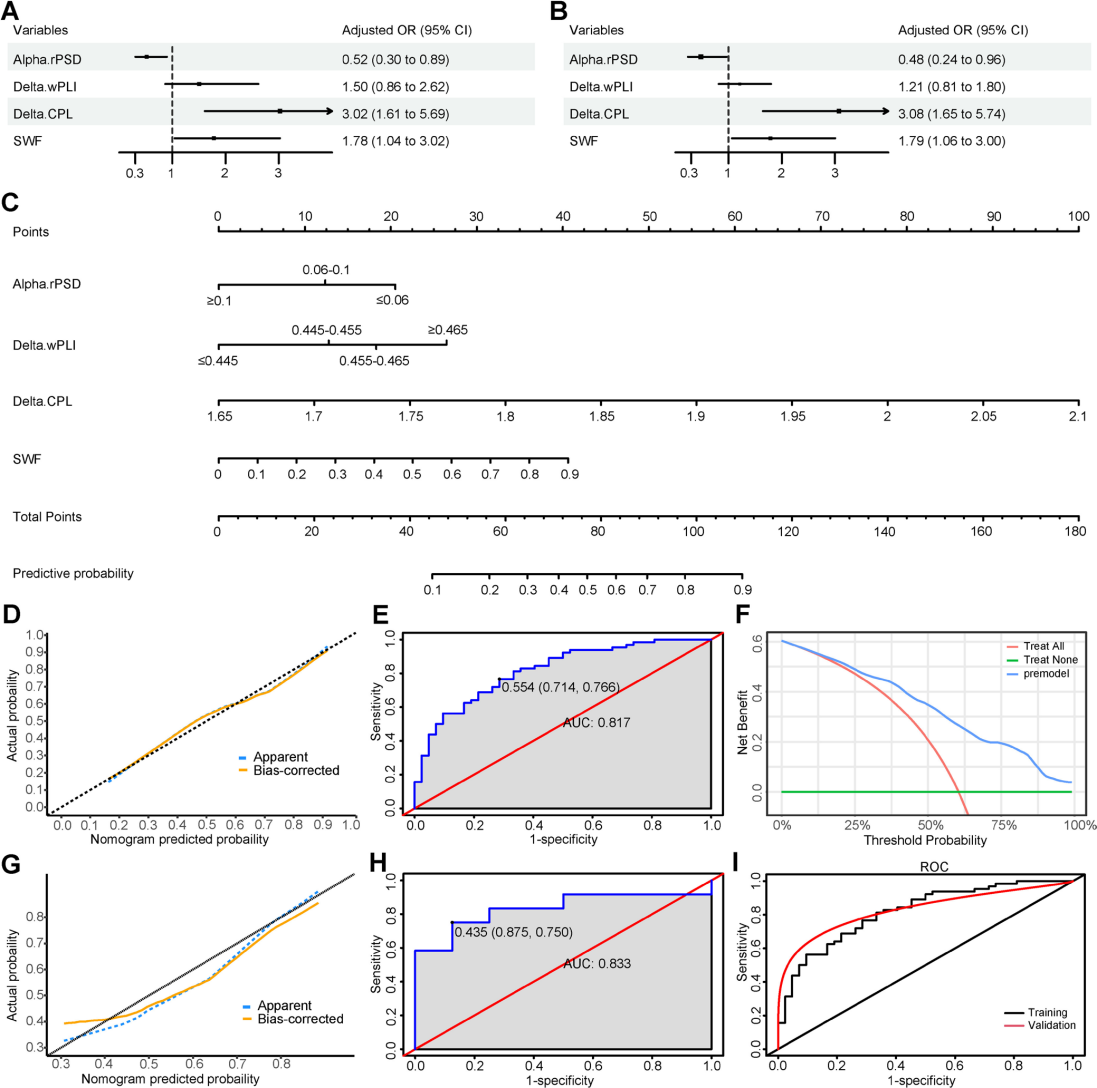
Construction and performance evaluation of the prediction model. (A) Forest plot based on multivariate logistic regression analysis of four factors in the form of continuous variables. Odds ratio (OR) values for continuous variables were standardized. The adjusted OR value was calculated based on the per‐standard deviation (SD) increase in variables. (B) Forest plot based on multivariate logistic regression analysis of four factors in the form of categorical variables and continuous variables. Relative power spectral density (rPSD) and weighted phase lag index (wPLI) were converted into categorical variables based on interquartile range. (C) Nomogram for prediction of developing EE‐SWAS in children with initial SeLECTS. Characteristic path length (CPL) was calculated by computing the area under the curve (AUC) across different sparsities (sparsity range 0.1–0.5, step length 0.05) and further normalized to obtain its true value. To use the nomogram, the scores for each factor on the Point axis are calculated and summed to obtain the total points, then a vertical line is drawn downward from the Total Point axis to locate the Probability axis and derive the corresponding predictive probability. (D) Calibration curve of the nomogram in the training cohort. The black diagonal line represents a perfect prediction, and the orange line represents bias correction by bootstrapping (*B* = 1000 replications), indicating that the actual probability of developing EE‐SWAS is generally consistent with the nomogram‐predicted probability. (E) Receiver operating characteristic (ROC) curve of the nomogram in the training cohort. (F) Decision curve analysis (DCA) of the nomogram in the training cohort. The red line represents the assumption that all children develop EE‐SWAS, and the green line represents the assumption that no children develop EE‐SWAS. The blue line represents the nomogram created with four factors which showed a higher net benefit value, suggesting the model's good utility in clinical practice. (G) Calibration curve of the nomogram in the external validation cohort. (H) ROC curve of the nomogram in the external validation cohort. (I) Comparison of the area under the curve (AUC) of ROC curves between the training cohort and the external validation cohort. External validation showed that the model still had good predictive performance. Abbreviations: AUC, area under the curve; CI, confidence interval; CPL, characteristic path length; OR, odds ratio; RPSD, relative power spectral density; SWF, spike–wave frequency; wPLI, weighted phase lag index.

After multivariate analysis (Figure 2A,B for multivariate regression analysis results), four factors were retained in the final model: SWF, relative PSD in the alpha band, wPLI in the delta band, and CPL in the delta band (Two continuous variables, relative PSD in the alpha band and wPLI in the delta band, were converted to categorical variables when constructing the final model). The VIF values ranged from 1.056 to 1.194 (no VIF > 10), indicating no collinearity among the variables. The nomogram and ROC curve of the training cohort are shown in Figure 2C,E, respectively. The model's AUC was 0.817 (95% CI = 0.736–0.898). Internal validation using the bootstrap method demonstrated a good predictive ability with an AUC of 0.816 (95% CI = 0.738–0.898). Both the calibration curve (Figure 2D) and the Hosmer‐Lemeshow test (*p* = 0.845) indicated a good fit. The DCA curve (Figure 2F) showed a higher net benefit value of the prediction model, suggesting the model's utility in clinical practice. The model was externally validated in 20 children who met the inclusion criteria, and it also had good discrimination, with an AUC of 0.833 (Figure 2H). The model for the validation cohort similarly demonstrated acceptable fit (Figure 2G).

## 4 Discussion

It is well‐recognized that some patients with SeLECTS undergo encephalopathic transformation as the disease progresses. Given the poor neurocognitive outcomes associated with EE‐SWAS, there is an urgent need for early predictors to indicate the emergence of EE‐SWAS. Recent studies have attempted to address this issue, but due to limitations in indicator selection and sample size, no successful models have yet been developed [36, 37, 38]. General clinical features lack effective predictive value for the encephalopathic transformation of SeLECTS. Our study indicated that clinical features were not significantly correlated with the progression to EE‐SWAS. Previous views suggested that sodium channel blockers used in SeLECTS may increase the risk of deterioration and induce encephalopathic transformation [39, 40]. However, this study found no potential correlation between specific anti‐seizure medication choices and the development of EE‐SWAS. Although epilepsy‐related features do not predict the progression of SeLECTS to EE‐SWAS, we observed the potential predictive value of early EEG and focused on analyzing several quantitative indicators. After multivariable logistic regression, four factors were retained in the final prediction model. Our model demonstrated good discrimination with an AUC of 0.817 (95% CI = 0.736–0.898), indicating up to 80% accuracy in predicting the occurrence of encephalopathic transformation in SeLECTS.

In a prospective study by Massa et al. [41], the risk of neurocognitive impairment in patients with SeLECTS was significantly associated with six distinctive interictal EEG patterns: intermittent slow‐wave focus, multiple asynchronous spike–wave foci, long spike–wave clusters, generalized 3‐c/s “absence‐like” spike–wave discharges, conjunction of interictal paroxysms with negative or positive myoclonia, and abundance of interictal abnormalities during wakefulness and sleep. Therefore, qualitative EEG characteristics may have potential suggestive effects for indicating the atypical evolution of SeLECTS. We analyzed common qualitative EEG indicators and found no clear correlation between initial abnormal EEG features and the development of EE‐SWAS. Previous studies have reported the potential predictive value of focal slow‐wave activities, Rolandic multiple spikes, and non‐Rolandic discharges in encephalopathic transformation [36, 37], and our results do not support these findings. Compared to independent spikes, poly‐spikes can be interpreted as more frequent spike firing, which may adversely affect cognitive development [42]. Our results showed that the proportion of children with multiple spikes was higher in those undergoing encephalopathic transformation (FDR‐corrected *p* = 0.313), suggesting the potential predictive role of multiple spikes, which needs to be validated in larger cohorts. Gregory and Wong [43] found that children with dipole discharges were less likely to exhibit developmental delays and learning difficulties compared to those with non‐dipole discharges, implying that non‐dipole discharges may represent a non‐benign functional focus potentially associated with encephalopathic transformation. However, our study did not observe this correlation.

Our study found that there was no difference in SWI on initial EEG between the groups, but that SWF was associated with the presence of EE‐SWAS. Over time, children with higher spike frequencies are more likely to develop EE‐SWAS. Neurodevelopmental regression in EE‐SWAS is highly correlated with the ESES pattern on EEG. Traditionally, ESES is defined as constant spike activity with SWI > 85%, although significant regression may also occur with a lower SWI (> 25%). Most children with EE‐SWAS exhibit epileptiform activity occupying > 50% of NREM sleep [44]. Initially, the EEG pattern of frequent discharges may not yet reach the level of constant epileptiform activity necessary for an ESES diagnosis, nor is it sufficient to cause neurodevelopmental abnormalities. However, a higher SWF implies more frequent spike discharges, which may adversely affect neurodevelopment through chronic damage. Interictal spike activity can disrupt sleep architecture, negatively affecting brain plasticity and memory consolidation, thereby impairing cognitive performance [42]. When occurring in a pattern of frequent discharges, this accumulative effect can lead to eventual encephalopathic transformation. It is reasonable to extrapolate that children with initially higher SWF are at a higher risk of deteriorating EEG patterns, and despite initial neurocognitive normalcy, they are likely to experience developmental regression as discharges increase, leading to a diagnosis of EE‐SWAS. SeLECTS and EE‐SWAS are phenotypically distinct epilepsies within the same continuous spectrum, and spike activity activation might be an endophenotypic feature shared across the spectrum [45]. The initial pattern of frequent discharges should not be simply viewed as an early stage of EE‐SWAS. We tend to believe that spike frequency reflects inherent differences in phenotype severity that are present from the initial stage.

Spectral power analysis converts time‐domain signals into frequency domain signals, allowing the quantification of spectral power by frequency bands [46]. In a MEG study by Li et al. [47], compared with children with typical SeLECTS (SWI < 50%), patients with mild SWI (50% ≤ SWI < 85%) exhibited an enhanced PSD in the delta band and attenuated PSD in the alpha band of the bilateral posterial cingulate cortex (PCC), and PSD of the PCC in the alpha band showed good accuracy in distinguishing between mild SWIs and typical SeLECTS. Similarly, our study showed lower relative PSD in the alpha bands in children undergoing encephalopathic transformation compared to those who did not. PSD is related to the balance between neuronal excitation and inhibition, reflecting the activity of neuronal populations firing synchronously in specific frequency bands [48, 49]. Differences in power indicate changes in neuronal synchrony or the number of neurons oscillating synchronously [50]. This may be related to altered extracellular space and ionic environments around neurons, leading to cortical hyperexcitability [51, 52]. This hyperexcitability does not always reach the threshold for inducing spike activity, but differences in epileptic susceptibility can be reflected in the PSD [53]. Notably, more spike‐and‐slow‐wave complexes in the EE‐SWAS group may contribute to higher power in the delta band. After multivariate analysis, only the power difference in the alpha band was retained as a predictor in the final model, which was consistent with the findings of Li et al. [47]. Overall, our findings suggest that the presence or absence of encephalopathic transformation is associated with power differences at the early stage of onset, implying differences in the underlying neurobiological basis.

In our study, we found that encephalopathic transformation was associated with increased global wPLI values in the delta bands. It is commonly thought that lower connectivity is associated with cognitive and behavioral abnormalities [54]. However, functional connectivity networks in the context of epilepsy are more complex. Aberrant hyper‐connectivity may impair the brain's ability to regulate activity in response to higher cognitive demands [55]. High phase locking in multiple brain regions leads to over‐synchronized neuronal firing, creating a pathological state of over‐coupling that likely results in diminished information transfer and ineffective interregional communication [56]. A study by Mott et al. [57] showed that EEG coherence was significantly higher in children with ESES compared to children with normal neurodevelopment, consistent with our findings.

After multivariate analysis to exclude confounding factors and interactions between variables, a positive correlation was observed between wPLI values in the delta band and EE‐SWAS outcomes. Considering that phase synchronization‐based metrics were used to calculate FC, frequent and highly synchronized spike waves in the delta band tend to have higher phase synchrony, affecting connectivity. In contrast, the results of further graph‐theoretical analysis may more accurately approximate the physiological significance of functional connectivity. Choi et al. [58] found that the mean CPL value was significantly longer for SeLECTS patients than for the disease‐free controls in the lower alpha band, indicating that the global integration of connectivity was reduced in SeLECTS; while our study found that SeLECTS children with encephalopathic transformation had longer CPL values in the delta band than those without. CPL, a measure of integration in the brain, reflects lower efficiency and worse global information communication when increased [59]. In addition, differences in the localization, lateralization and synchronization of discharges may have an impact on the connectivity of individuals, but our final results were presented at the group level, and there was no difference in these factors between groups. Therefore, the difference of the FC metrics was meaningful. Overall, our findings suggested that children who evolve to EE‐SWAS may exhibit poorer brain development and potential global dysfunction at the onset of SeLECTS. It is important to note that this study aimed to develop EEG markers through FC analysis to predict the evolution of SeLECTS, rather than to emphasize a causal link between connectivity and EE‐SWAS. More extensive research is needed to elucidate the underlying mechanisms of the relationship between functional brain networks and the encephalopathic transformation of SeLECTS.

## 5 Limitations and Directions

The main limitation of this study stems from its retrospective design. Firstly, not all patients underwent genetic testing, which may have missed the potential predictive value of genetic information. On the one hand, our limited results found a higher proportion of pathogenic or likely pathogenic gene mutations in the EE‐SWAS group; on the other hand, there may be a correlation between specific genes such as SCN2A and EE‐SWAS [60]. Therefore, it is valuable to perform a comprehensive genetic test to identify the potential pathogenic genes of EE‐SWAS. Secondly, for children initially diagnosed with SeLECTS, the neurocognitive status of a small proportion was based on patient complaints and clinician assessments rather than rigorous neuropsychological testing. Thirdly, we may have missed some factors with potential predictive value, such as high‐frequency oscillations (HFOs) in EEG and MRI‐based radiomics signatures (thalamic volume, cortical thickness, etc.), which could not be analyzed further due to limitations in the raw data. In addition, the qEEG analysis in our study was based on a single diagnostic EEG, and the indicators of SWI and SWF were calculated based on manual visual inspection. These study deficiencies may lead to a degree of unreliability and become potential sources of bias. Furthermore, as mentioned before, the localization, lateralization, and synchronization of discharges may affect the individual‐level FC results to some extent. It would be beneficial to conduct a paired study of these qualitative characteristics to eliminate the influence of potential confounding factors on the results. Finally, the selection of different methods for calculating power and FC metrics may introduce slight variations in the final results. It is worth noting that our study included typical SeLECTS children and excluded those with an initial SWI > 50%. In fact, some patients with EE‐SWAS do have a high SWI at the onset of SeLECTS, and the choice of the 50% threshold may cause a certain deviation between our results and the actual situation. Further research is needed for SeLECTS population with a high initial SWI. Future prospective multi‐center studies with larger sample sizes and comprehensive genetic tests are needed to establish more accurate and efficient prediction models by combining clinical manifestations with EEG and MRI features. More research is still needed to clarify the complex interrelationships between spike–wave discharges, EEG metrics, and EE‐SWAS, and to reveal the potential mechanisms of encephalopathic transformation in SeLECTS.

## 6 Conclusion

In children with SeLECTS, early EEG features such as SWF, relative PSD, wPLI, and CPL were significantly associated with the presence of EE‐SWAS during the disease course. This suggests that children undergoing encephalopathic transformation may have different disease characteristics and pathogenesis from the outset compared to those who do not. By introducing early quantitative EEG indicators, we successfully constructed a model to predict the evolution of EE‐SWAS in SeLECTS, thereby aiding early clinical diagnosis and timely intervention for EE‐SWAS.

## Author Contributions

Zimeng He and Baomin Li contributed to the conception and design of the study. Zimeng He, Linghui Zhu, Zaifen Gao, Xiaofan Yang, Yumei Li, Xiaoyu Zhao, Lili Tong, and Guijuan Jia contributed to the acquisition, analysis and interpretation of data. Zimeng He, Dongqing Zhang, and Baomin Li contributed to drafting the article or revising it critically for important intellectual content.

## Ethics Statement

We confirm that we have read the Journal's position on issues involved in ethical publication and affirm that this report is consistent with those guidelines.

## Conflicts of Interest

The authors declare no conflicts of interest.

## Data Availability

The original data that support the findings of this study are available from the corresponding author upon reasonable request.
